# Forensic Toxicological and Medico-Legal Evaluation in a Case of Incongruous Drug Administration in Terminal Cancer Patients

**DOI:** 10.3390/toxics9120356

**Published:** 2021-12-16

**Authors:** Pascale Basilicata, Pasquale Giugliano, Giuseppe Vacchiano, Angela Simonelli, Rossella Guadagni, Angela Silvestre, Maria Pieri

**Affiliations:** 1 Legal Medicine Section, Department of Advanced Biomedical Science, University of Naples “Federico II”, 80131 Naples, Italy; pbasilic@unina.it (P.B.); angela.simonelli@unina.it (A.S.); r.guadagni@unina.it (R.G.); angela.silvestre@unina.it (A.S.); 2 Legal Medicine Section, AORN “Sant’Anna e San Sebastiano” Caserta, 81100 Caserta, Italy; dottgiugliano@tiscali.it; 3 Department of Law, Economics and Mathematical Methods, University of Sannio, 82100 Benevento, Italy; vacchiano952@gmail.com

**Keywords:** deep sedation, drug interaction, midazolam, morphine, palliative care, terminal patient management

## Abstract

Background: In most cases, palliative care is prescribed to adults diagnosed with cancer. The definition of the most suitable therapy for an effective sedation in terminal cancer patients still represents one of the most challenging goals in medical practice. Due to their poor health, the correct dosing of drugs used for deep palliative sedation in terminal cancer patients, often already on polypharmacological therapy, can be extremely complicated, also considering possible drug-to-drug interactions that could lead to an increased risk of overdose and/or incongruous administration with fatal outcomes. The case of a terminal cancer patient is presented, focusing on the “adequacy” of administered therapy. Materials and Methods: A young male, affected by Ewing sarcoma, attending a palliative care at his own home, died soon after midazolam administration. Toxicological and histological analyses were performed on body fluids and organ fragments. Results and Discussion: Morphological reliefs evidenced a neoplastic mass, composed of lobulated tissue with a lardy, pinkish-gray consistency, extending from the pleural surface to the lung parenchyma, also present at the sacrum region (S1–S5), at the anterior mediastinum level, occupying the entire left pleural cavity, and infiltrating the ipsilateral lung. Metastatic lesions diffused to rachis and lumbar structures. The brain presented edema and congestion. Toxicological analyses evidenced blood midazolam concentrations in the range of 0.931–1.690 µg/mL, while morphine was between 0.266 and 0.909 µg/mL. Death was attributed to cardiorespiratory depression because of a synergic action between morphine and midazolam. The pharmacological interaction between midazolam and morphine is discussed considering the clinical situation of the patient. The opportunity to proceed with midazolam administration is discussed starting from guidelines recommendation. Finally, professional liability outlines are highlighted.

## 1. Introduction

Pain has always been a source of concern for mankind and the subject of a ubiquitous commitment to understand and control it. Remedying suffering and offering relief to the patient mean improving their quality of life and, in general, the quality of the health care provided. When the patient’s health conditions worsen and curation is no longer possible, palliative care becomes the only feasible approach, the purpose of which changes as the patient’s health conditions change [1]. If the goal is to ensure or improve quality of life, understanding the relationship between comfort and function maintenance is crucial to correctly use sedation: if the patient’s condition allows it, sedation is an unintentional consequence of therapy, while during the last moments, it represents the only therapeutic possibility [1]. Since 2002, palliative care has been included in the European Charter of Patients’ Rights [2], as point 11 “Right to Avoid Unnecessary Suffering and Pain.” De Graeff and Dean recommended the use of palliative sedation therapy for “specific sedative medications to relieve suffering from refractory symptoms by a reduction in patients’ consciousness,” while refractory symptoms are the ones “for which all possible treatment has failed, or it is estimated that no methods are available for palliation within the time frame and the risk-benefit ratio that the patient can tolerate” [3].

In March 2010, a legislative decree was issued in Italy focusing on palliative care and pain therapy, also introducing the at-home sedation [4]. The Italian Society of Palliative Care underlines the opportunity for the physician to establish an efficient communication with both the patients and the relatives [5]. With respect to the deep sedation, the Italian Commission on Bioethics [6] suggested the following eligibility criteria for access to deep sedation: a patient with an incurable disease in an advanced stage, imminent death, the patient’s consent, the presence of acute terminal events or symptoms refractory to treatment causing intolerable suffering to the patient. In a recent review on clinical aspects related to palliative sedation, Arantzamendi and colleagues [7] evidenced that “refractory symptoms most frequently reported were the delirium (41–83%), the pain (25–65%) and dyspnea (16–59%)”; moreover, psychological, and existential distress occurred in 16–59% of patients, with midazolam as the most administered drug.

Terminal cancer patients are among the most fragile and critical and the definition of the most suitable palliative care is still an open challenge: the precarious health conditions that these patients generally present in the terminal stages of their lives, together with therapies (often polytherapy) already prescribed, contribute to complicate the choice of the best approach to ensure adequate quality of life. If sedation must be ensured for a short time period, blood pressure, oxygen saturation, and respiratory rate have to be monitored, as well as the degree of sedation established according to the RAMSEY scale; therapy needs the presence of sanitary personnel during the first 10–15 min.

Finally, palliative care therapy itself can involve the use of several active principles, for which the clinician must carefully evaluate the possible drug-to-drug interactions considering the specific patient’s health conditions.

Opiates, mostly morphine, in combination with midazolam and haloperidol, are widely used for symptom control [8]. Drug-to drug interactions occurring in polytherapy involving the simultaneous administration of morphine and benzodiazepines are well documented [9], but additional considerations must be pointed out to approach the correct management of terminal cancer patients requiring the simultaneous administration of such drugs to ensure adequate pain control and/or sedation. All pharmacokinetic (Pk) phases may be subject to nonnegligible variations in terminal cancer patients, resulting in drug bioavailability sensibly divergent from levels that are predictable on the basis of administered doses and “normal” Pk parameters, as determined from studies on healthy volunteers [10]. A modification in metabolism deriving from the advanced illness can modify drugs’ pharmacokinetics as well [11]. Franken and colleagues [12] reviewed this item, evidencing that deep modifications in drug absorption rate, bioavailability, metabolism itself can occur in terminal cancer patients, due to changes in hepatic functions or liver blood flow, gastrointestinal problems or symptoms such as nausea and vomiting commonly registered, tissue blood perfusion, and subcutaneous fat content. This last aspect may determine an increased absorption rate, resulting in higher peak concentrations with respect to results of studies performed on healthy volunteers. A continuous and accurate monitoring of the real patient’s metabolism is mandatory for a correct and safe pharmacological therapy in terminal cancer subjects approaching the last days of life, and modifications in administered therapy involving the introduction of new drugs or a higher dosage can be decided only in light of a documented worsening of the patient’s conditions. It should also not be forgotten that the administered palliative therapy must be discussed and agreed with the patient, until their conditions allow it, or alternatively with relatives and/or legal representatives.

Within this item, the present paper presents the case of a terminal cancer patient who died at their own home in strict temporal relation with a midazolam administration. Toxicological data are discussed in view of guidelines and recommendations for a safe management of terminal cancer patients and possible professional liability.

## 2. Case Report

A young Italian male was diagnosed with Ewing’s sarcoma in the sacral region when he was 24 years old. Despite second- and third-line chemotherapy treatments, as well as radiotherapy, a chest recurrence of sarcoma presented after four years, with costal, pleural, pulmonary, and cardiac involvement. Due to his worsening health conditions and the severity of the painful symptoms, the young man began a treatment of palliative care in an Onco-Hematology Department, including morphine (0.8 mL/min) for analgesia. As the disease worsened, he decided to continue with palliative care at his home and was entrusted to the Territorial Service for Integrated Home Care. The following therapy was prescribed: oxygen, 3 L/min; paracetamol, 1 g iv, at occurrence; morphine, 100 mg at 1.5 mL/h, with continuous infusion; morphine, 3 mg/3 mL infusion in physiological solution, at occurrence; no other drugs were prescribed for patient sedation. The young man died after three days from the arrival at home. Although the clinical diary did not report any worsening of the condition of the young man that justified the implementation of analgesia, empty bottles of midazolam were found in the bedroom. A total of eight empty ampoules (six of 5 mg and two of 15 mg) were found in the bedroom, thus suggesting a possible 60 mg midazolam administration. The parents reported that the physician proceeded with the administration of midazolam (not authorized in Italy for home treatment), moving away immediately after. The parents called the emergency medical services, who reported the death to the Prosecutor Office (P.O.). Both autopsy and toxicological analyses were performed, in an attempt to elucidate the exact cause of death (natural or induced by incongruous drug administration), as well as to verify eventual professional liabilities.

## 3. Material and Methods

Certified standard solutions of drugs of abuse used for confirmation analysis in gas chromatography/mass spectrometry (GC/MS) were from Cerilliant-Merck (Milan, Italy), *N*,*O*-bis (trimethylsilyl) trifluoroacetamide (BSTFA) derivatizing agent from Acros (Morris Plains, Morris Count, NJ, USA), and HPLC-grade solvents from Carlo Erba (Milan, Italy).

Enzyme-Linked ImmunoSorbent Assay (ELISA) screening tests were performed on a Dynex-DSX system from Technogenetics (Chantilly, VA, USA), using forensic blood kits from Abbott for AMP/MAMP/MDMA, barbiturates, benzodiazepines, buprenorphine, cannabinoids, cocaine, fentanyl, ketamine, methadone, opiates, oxycodone, tricyclic antidepressants, and zolpidem.

GC/MS analyses were performed using an ISQ single-quadrupole mass spectrometer directly linked to a Trace1300 gas chromatograph equipped with a split-splitless autosampler Al1310, all from ThermoFisher (San José, CA, USA). Gas chromatographic separations were performed with a Rxi^®^-5MS (30 m × 0.25 mm × 0.25 µm) capillary column (Restek, Bellefonte, PA, USA). Data were processed using the Xcalibur software (version 4.0.27.13) from ThermoFisher.

Headspace gas chromatographic/mass spectrometric (HS-GC/MS) analyses were performed on an HP6890 series gas chromatographer provided with a HP7694E autosampler and a 5973 single-quadrupole mass spectrometer (Hewlett-Packard, Palo Alto, CA, USA); chromatographic separation was accomplished by a CP PorabondQ capillary column (Varian, Agilent, Santa Clara, CA, USA), and data were analyzed using the MSD Chemstation software (D.02.0.275 version) from Agilent Technologies (Santa Clara, CA, USA).

### Toxicological Analysis

Biological fluids (left jugular vein, cardiac, portal vein and aorta vein blood, urine, and bile) and organ homogenates (from brain and liver) were used for toxicological analyses; femoral blood, usually used for post-mortem toxicological analyses [13], was not collected, due to body conditions. ELISA screening tests were initially performed on portal vein blood, after dilution (1:10, v:v) of a proper sample aliquot with bidistilled water, according to the manufacturer’s specifications. All “nonnegative” results were verified with specific conformation analyses performed on all biological matrices by GC/MS, after appropriate deuterated internal standard addiction and proper purification through solid-phase extraction and eventual derivatization, according to analytical procedures previously validated [14,15]. For GC–MS analyses, all samples were acquired in both *full scan* and selected-ion monitoring mode (GC/MS-SIM). The eventual presence of ethyl alcohol or any other volatile chemicals was also verified, by analyzing portal vein blood by HS-GC/MS.

**Figure 1:**
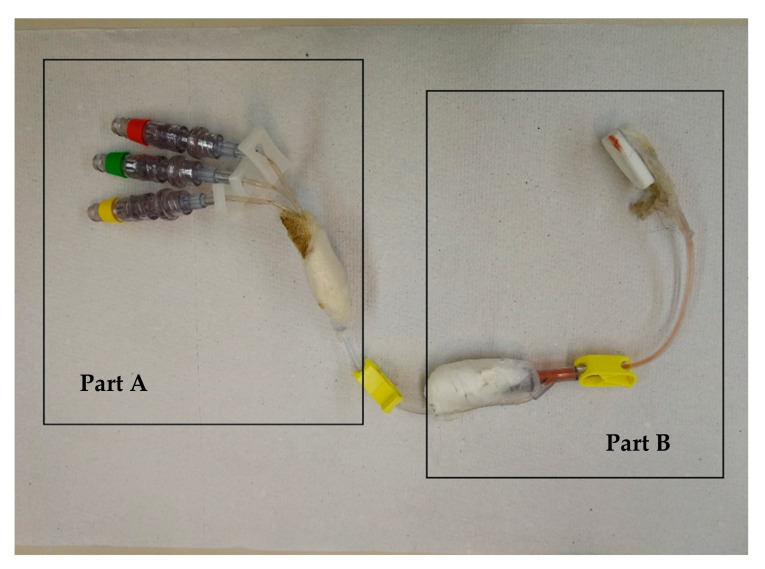
Central venous catheter recovered in deceased’ under-right clavicular region.

Toxicological analyses were also performed on liquid residues recovered from the central venous catheter present in the under-right clavicular region of the deceased; solutions of three infusion lines (see Figure 1, part B) and of the final tract (see Figure 1, part A) were analyzed in GC/MS.

## 4. Results

### 4.1. Autopsy

The autopsy highlighted the presence of a central venous catheter placed in the right clavicular vein, connected distally with three different infusion lines (red, green, and yellow, see Figure 1). In the left hemithorax, a serious and extensive neoplasm was present. The neoplasm occupied much of the pleural cavity and the left lung had collapsed so that the parenchymal area was highly limited. The neoplastic tissue originated from the ribs of the left hemithorax, showing a thin and pasty tissue from the second and up to the twelfth arc, to testify that the sarcomatous localization affected extensively the costal bony plane. Finally, the pelvis skeleton showed macroscopic morphological findings of the original sarcomatous lesion, which also affected the last lumbar vertebrae (4th on 5th).

### 4.2. Histological Examinations

Histological investigations confirmed at the left hemithorax the presence of multiple locations of Ewing’s sarcoma with large areas of necrosis and hemorrhage and bilateral bronchopneumonitis outbreaks.

A neoplastic localization of Ewing’s sarcoma was also highlighted at the level of the trachea. Areas of fibrosis were evidenced on the myocardial tissue, reasonably framed as outcomes of chemotherapy and radiotherapy treatments suffered by the patient.

### 4.3. Toxicological Analysis

Toxicological screening tests performed on an aliquot of the portal vein blood resulted as “nonnegative” toward benzodiazepines and opiates, and the datum was confirmed by GC/MS. All sampled biological matrices resulted as positive toward morphine; midazolam was detected in all samples but liver homogenate; GC/MS *full scan* analyses resulted as negative toward hydroxymidazolam. Quantification was performed in GC/MS-SIM; the results are presented in Table 1; morphine quantitative data refer to total morphine, as samples underwent acidic hydrolysis before purification.

The results of toxicological analyses performed on fluids and organ homogenates evidenced positivity toward midazolam and morphine. Midazolam concentrations varied within the range from 0.9 µg/mL (portal vein blood) to 1.7 µg/mL (aorta vein blood); the benzodiazepine was also detected in brain homogenate (1.3 µg/g), while bile, urine, and liver resulted as negative. All analyzed biological matrices resulted as positive to morphine, with concentrations from 0.3 µg/mL (aorta vein blood) up to >0.8 µg/mL (portal vein blood, urine, and bile); the brain and liver presented morphine concentrations of 0.9 ng/g and >0.8 µg/g, respectively.

Results on morphine are in line with pharmacological therapy prescribed to the patient. He presented intense painful symptoms, as the neoplasm infiltrated a large part of the pelvis skeleton, the last lumbar vertebral metamers, numerous costal elements of the left hemithorax in one on the entire pleural surface, and almost all the ipsilateral lung. The prescribed analgesic therapy provided for continuous morphine infusion (and paracetamol at occurrence), justified by a picture of certainly very intense pain, well documented in the medical record.

Different considerations can be made with respect to midazolam concentrations. Regarding the quantitative levels of the drug determined in the different biological matrices, it must be underlined as post-mortem data cannot be simply interpreted by comparison with in vivo therapeutic concentrations. Modifications occurring immediately after death (incomplete drug distribution at the time of death, release from the binding site, passive diffusion) account for significative variations between ante- and post-mortem drug levels [16,17]. Comments on thanatological data must be done with respect to post-mortem studies. Midazolam concentrations highlighted in the case presented here are from 4.2 to 7.7 times higher than the literature datum. Data on midazolam overdoses refer mostly to erroneous administrations by sanitary personnel [18] or to ampoule labeling errors [19] as a major source of iatrogenic injury in hospitalized patients, although they are rarely associated with patients’ death [19]. In one case of a “rare midazolam overdose” administered to the victim through an adulterated drink, Wang et al. reported a benzodiazepine concentration, measured in the cardiovascular system, of 0.22 µg/mL [20].

Toxicological analyses performed on solution residues recovered from the central venous catheter evidenced the presence of midazolam in Part A-yellow line and Part B solutions.

## 5. Discussion

Despite being provided in palliative sedation protocols, simultaneous administration of a sedative-hypnotic benzodiazepine in a terminal cancer patient already under continuous morphine infusion must be carefully evaluated. When administered to healthy patients, sedative-hypnotics induce effects on respiration comparable to those recorded during natural sleep even at hypnotic doses [21]. Conversely, the administration of these drugs in patients with obstructive pulmonary diseases can induce significant respiratory depression, even at therapeutic doses. In such patients, therefore, it becomes crucial to control the occurrence of possible additive effects, following the simultaneous administration/intake of other drugs characterized by depressive action on the central nervous system. The additive Central Nervous System depression occurring when benzodiazepines are taken together with alcoholic beverages, analgesics, opioids, anticonvulsants, phenothiazines, and other sedative-hypnotics is well documented in the literature [21]. In particular, the simultaneous administration of morphine and sedative-hypnotics has the effect of strengthening the depression of the central nervous system, regarding the enhancement of respiratory depression [21]. In the case presented here, the patient had limited respiratory function following the extension of the neoplastic disease to the entire left hemithorax. Consequently, the administration of an active principle known to induce CNS depression required an adjusted dose to clinical conditions and the careful monitoring of the patient’s clinical evolution and could only be justified to remedy a significant worsening of his painful symptoms.

Midazolam is classified as a “short-acting” drug, with a rapid onset of the pharmacological effects [11], further enhanced in the case presented here by the subcutaneous injection. Administration by intravenous injection bypasses the absorption phase, normally representing the slowest step directly determining the time required for the beginning of pharmacological effects. When administered intravenously, the clinical onset is determined exclusively by the time necessary for the drug to reach the brain through the bloodstream starting from the injection point and by the time required for the passive diffusion of the active principle across the blood–brain barrier: this process requires typically 15 s to 5 min, regardless of the intravenous access used, and *“depending on the size of the dose, the particular pharmacologic response, and the patient’s sensitivity”* [22].

Toxicological findings showed the presence of midazolam in part A (yellow line) and part B solutions recovered from the central venous catheter present on the body, confirming the hypothesis of benzodiazepine administration through such a device. In view of its position on the body (under-right clavicular vein), the positivity found in all blood samples attests the effective midazolam distribution. In this regard, particularly relevant is the positivity of the blood sample from the portal vein: if the midazolam did not have time to distribute, such a sample would have been substantially negative. Moreover, considering the positivity of the brain homogenate sample, the drug had the opportunity to exert its pharmacological effects before the patient’s death. Negative results obtained for bile, liver, and urine toward midazolam strongly supported the hypothesis of a strict correlation between drug administration and death. Such an aspect is of great relevance to assess the sanitary management, as discussed below.

In the case presented here, the midazolam administered dose, estimated by the empty ampoules (six of 5 mg and two of 15 mg) found in the deceased’s bedroom, was about 60 mg by multiple intravenous injections performed in a short time sequence, therefore comparable to a single injection. Such an administered dose results as higher than dosages reported in the literature for palliative sedation of terminal cancer patients. In their revision of the literature, De Graeff and Dean reported the use of different drugs (both in terms of active principle and dosage) among countries, but, generally, midazolam is the sedative of choice, due to its several advantages, such as short half-life, few side effects, anxiolytic, antiepileptic, and muscle relaxant properties, apart from sedative effects [3]. The administered mean concentration in the literature reviewed by De Graeff and Dean varied in the range of (22–70) mg/24 h, while the median dose was within (30–45) mg/24 h [3]. In a 2011 retrospective cross-sectional study in a home cohort, Calvo-Espinos et al. [23] reported similar dosages: 35 patients treated at home were administered with a mean midazolam dose of 40 mg during the last day of life. Higher dosages were reported by Alonso-Babarro et al. [24] in a retrospective review on palliative therapies prescribed at home to terminal cancer patients between 2002 and 2004: 27 out of 29 patients received midazolam for palliative sedation, with a mean dosage in the last day of life of 74 mg; two patients required the administration of levomepromazine (mean dosage: 125 mg during the last 24 h). Porzio et al. [25] used a multi-step midazolam-based therapy to achieve sedation in patients presenting delirium (13 subjects) or dyspnea (3 subjects). Dosages of 1 mg/h were initially administered; in one third of treated patients, the dosage was doubled with the addition of chlorpromazine and promethazine to maintain a deep and effective sedation. Prommer recently published a review article on midazolam as an *essential palliative care* drug [26]: literature dosages varied in the range of (15–60) mg/day for the sedation of uncontrolled symptoms in a South Africa hospice [27], (23–58) mg/day in an Italian study on terminal cancer patients assisted at home [28], and up to 79 mg/day administered in an Israeli hospice [29]. As evident, all cited literature refers to therapies administered gradually during the 24 h, with a careful titration of the sedative dose *to the relief of symptoms and the distress it* causes [3]. Moreover, all changes in therapy—both in terms of the administered active principle and dosage increase—must be a consequence of a worsening in the patient’s conditions, which must be well documented in the clinical care diary [3]. These recommendations were completely disregarded in the case presented here, as the physician proceeded with a 60 mg midazolam administration in a terminal cancer patient, presenting a well-documented reduction in lung function due to cancer extension (involving the entire left hemithorax and pleural surface and almost the entire left lung) and who was already on continuous morphine infusion therapy. Moreover, no indication of a worsening in patient’s conditions that could justify and/or suggest the need for further sedation through benzodiazepine administration was reported on the clinical care diary. Finally, the physician left the patient’s house immediately after midazolam administration, thus failing to comply with the obligation to monitor the evolution of his condition. Really, the positivity itself toward midazolam in a patient treated at home is of great concern. In Italy, such benzodiazepine is unavailable for extra hospital use, and its administration for palliative sedation of a terminal patient treated at home requires the authorization by hospital-home teams [28]. Such an authorization was not present, nor even required, in the case discussed here, thus representing the first critical aspect that negatively characterizes the healthcare professional’s conduct. The sanitary personnel decided (i) to implement the therapy by adding midazolam, (ii) to administer a dose exceeding ranges normally applied in palliative therapies, (iii) to proceed without a clear and well-documented worsening of the patient’s conditions; moreover, he moved away from the patient’ house immediately after drug administration without any control of the patient clinical evolution: such actions configure precise profiles of severe negligence. Based on the results of toxicological analyses, and of the autopsy and pathological examination, the Prosecutor Office referred the physician who proceeded with midazolam administration for murder. Euthanasia is not allowed in Italy and the so-called “consented murder” can only be allowed if the terminally ill patient has clearly expressed the will to die and is unable, due to his infirmity, to commit suicide [30]. In fact, article 580 of the Code of Criminal Procedure (which punishes assisted suicide) has been declared not entirely compliant with constitutional principles [30]. In the case presented here, the act could not be configured as consented murder, as the young man had never expressed the will to end his life, wishing only relief from the pain he suffered.

## 6. Conclusions

Pharmacological management of palliative sedation must be carried out with particular care, as it is always recommended to: (i) draw up an adequate clinical diary to report the therapeutic responses and possible side-effects progressively evaluated; (ii) gradually increase the drug’s dosages up to the desired sedation level; (iii) weigh the degree of sedation and any relative changes to therapy [3,31]. In general, sedation should be implemented at low initial doses, progressively increasing them until the degree of sedation is reached, aiming to control physical or mental symptoms. It is also necessary to safely conduct palliative sedation by monitoring its depth and symptom control, using tools such as recording vital parameters and measuring peripheral oxygen saturation. Of course, any variation in pharmacological therapy must take into account possible interactions with active principles already administered to the patient, as well as a pre-existing deficit and/or significative reduction in any function or organ that could be enhanced by the new drug, thereby compromising the patient’s life.

## Figures and Tables

**Table 1:** Morphine and midazolam concentrations detected in analyzed biological matrices.

Matrix	[Midazolam] (µg/mL)	[Morphine] (µg/mL)
left jugular vein blood	1.1	0.6
portal vein blood	0.9	>0.8
cardiac blood	1.3	0.7
aorta vein blood	1.7	0.3
urine	N.D.	>0.8
bile	N.D.	>0.8
brain	1.3 µg/g	0.9 ng/g
liver	N.D.	>0.8 µg/g

## Data Availability

Not applicable.
